# Supplementary material for: Neutrophils actively swell to potentiate rapid migration
Source: bioRxiv. 2024 Mar 28:2023.05.15.540704. Originally published 2023 May 16. Preprint. [Version 3] doi: 10.1101/2023.05.15.540704 (PMC10245588; doi:10.1101/2023.05.15.540704)
Supplement: 8 [file NIHPP2023.05.15.540704V3-supplement-1.pdf]

bioRxiv preprint doi: https://doi.org/10.1101/2023.05.15.540704; this version posted March 29, 2024. The copyright holder for this preprint (which was not certified by peer review) is the author/funder, who has granted bioRxiv a license to display the preprint in perpetuity. It is made available under aCC-BY 4.0 International license.

682 ## Supplementary Information

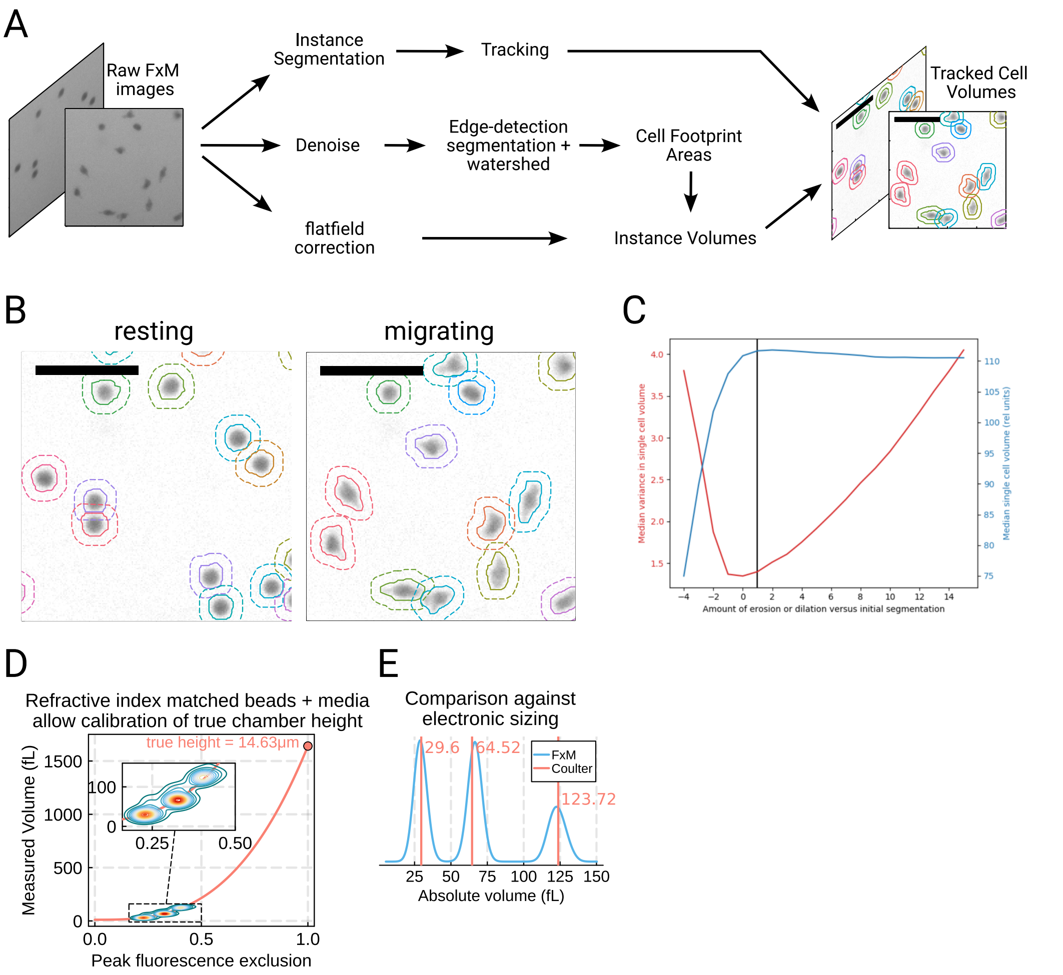

683

Figure 1—figure supplement 1. Details and validation of the Fluorescence eXclusion Microscopy pipeline

(**A**) Overview of the modified FxM pipeline used in this work. Raw FxM images are segmented into "seeds," which are then tracked. To identify cell boundaries, we use a custom algorithm that involves denoising the raw FxM followed by edge detection and watershedding that was nucleated at the seeds. Finally, to extract the volumes we apply minimal processing with a custom flatfield correction algorithm followed by extraction using the previously-determined cell boundaries. (**B**) Examples of resting (left) and migrating (right) cells segmented and tracked using the aforementioned pipeline. (**C**) Quantifying the signal-to-noise tradeoff with the edge detection. Overly conservative or overly liberal segmentation leads to non-optimal volume measurement. The chosen cutoff (black line) balances the noise (red) and measured volume (blue). This was computed on unstimulated cells. (**D**) Injecting beads into the FxM chambers in refractive-index media to minimize distortions allows for true calibration of chamber height. The contour plot of the per-bead max signal depth (i.e. height of the beads from FxM) vs the measured volume using FxM is well fit by the spherical volume formula (salmon line). The intersection of this line with 1.0 on the abscissa is equivalent to a sphere that completely fills the chambers and indicates the true height of the chamber. (**E**) Comparing calibrated FxM volumes of the beads (cyan) against the gold standard Coulter counter (salmon) shows good agreement.

bioRxiv preprint doi: https://doi.org/10.1101/2023.05.15.540704; this version posted March 29, 2024. The copyright holder for this preprint (which was not certified by peer review) is the author/funder, who has granted bioRxiv a license to display the preprint in perpetuity. It is made available under aCC-BY 4.0 International license.

Ranking	HUGO ID	Fold Change	FDR	# Good sgRNAs	Common Name
**1**	**FPR1**	**4.4617**	**0.00248**	**3**	**FPR1**
2	STAT3	2.8815	0.00248	4	
**3**	**SLC9A1**	**3.5013**	**0.00495**	**4**	**NHE1**
**4**	**PIK3CG**	**3.3557**	**0.05569**	**4**	**PI3Kγ**
5	MARS2	23.6178	0.06374	3	
6	NUP35	14.5253	0.06374	3	
7	SKA1	3.3554	0.06374	4	
**8**	**CA2**	**2.8157**	**0.09942**	**4**	**CA2**
9	CANX	3.966	0.09942	4	
10	COPS3	8.2426	0.10015	4	
11	RPS2	18.7498	0.07976	3	
12	TPR	9.6024	0.16455	4	
13	RFT1	26.2401	0.16455	2	
14	SETD1A	11.1409	0.16455	2	
15	TP53TG3C	11.2083	0.16455	3	
16	UHRF1	4.7194	0.16455	3	
17	THG1L	10.4411	0.16455	3	
18	MRPL20	10.8506	0.16455	4	
19	UTP11L	9.0882	0.16455	3	
20	TPI1	17.976	0.16455	3	
21	CT47A6	2.8505	0.16455	3	
22	ESCO2	11.9249	0.1982	4	
23	GRXCR2	2.8457	0.1982	4	
24	PARP12	21.181	0.1982	2	
25	NR1H2	39.6651	0.20894	2	
26	PPP2R2A	4.4472	0.20894	4	
27	SLC25A26	20.7941	0.20894	2	
28	GANAB	2.7762	0.20894	3	
29	MMGT1	22.231	0.24088	2	
30	TCEB2	9.1192	0.26382	4	
31	MRPL17	9.465	0.26382	3	
32	C1QB	2.7157	0.26382	4	
33	TMX3	3.0917	0.26382	4	
34	TOPBP1	14.0471	0.16455	3	
35	AP3M1	7.9696	0.26528	2	
36	MSMB	4.5751	0.26528	4	
37	WDR83	5.4812	0.26528	4	
38	MARCH7	6.5514	0.2937	2	
39	RNF111	2.9736	0.2937	4	
40	MOGS	3.5169	0.2937	3	
**100**	**SLC4A2**	**2.582**	**0.54636**	**2**	**AE2**

Table S1. Chemoattractant-induced swelling genome-wide CRISPR KO screen hits
Rankings determined using MAGeCK ***Li et al.*** (***2014***). Fold change is the median fold enrichment in the dense bin versus the other two bins of the functional guides. FDR is the false discovery rate of each gene given the distribution of negative control guides in the library. See Methods section for details.

bioRxiv preprint doi: https://doi.org/10.1101/2023.05.15.540704; this version posted March 29, 2024. The copyright holder for this preprint (which was not certified by peer review) is the author/funder, who has granted bioRxiv a license to display the preprint in perpetuity. It is made available under aCC-BY 4.0 International license.

Volunteer	Age	Sex
1	28	F
4	27	M
5	30	M
6	37	F
8	31	M
14	24	M
17	29	F
18	28	M
19	30	M
20	27	M

Table S2. Volunteer Demographic Information
Demographic information collected for the volunteer donors according to the Institutional Review Board-approved study protocol at the University of California - San Francisco (Study #21-35147)

HUGO gene name	Takara sgRNA ID	Guide Sequence (5′ -> 3′)	Internal ID
FPR1	sgFPR1_2	CTACAGTACCTGGTAAAACG	11
FPR1	sgFPR1_3	CTGACAGCAACGATGGACAT	12
SLC9A1	sgSLC9A1_2	TTTGCCAACTACGAACACGT	13
SLC9A1	sgSLC9A1_4	TGAGGAACAGGTCACACATG	15
PIK3CG	sgPIK3CG_1	ACTTAACCCTCTCACAGCAG	16
PIK3CG	sgPIK3CG_3	GAGAATACGTCCTCCACATG	18
CA2	sgCA2_2	TATGAGTGTCGATGTCAACA	19
CA2	sgCA2_4	TCACTGGAACACCAAATATG	20
SLC4A2	sgSLC4A2_2	ACCTGCCCCACATACCCACA	21
SLC4A2	sgSLC4A2_3	GAAGACGCAGGACCTGATAG	22
NegCtrl	Neg_Control_Human_0067	GGTAGGACCTCACGGCGCGC	23
NegCtrl	Neg_Control_Human_0084	GCTGTCGTGTGGAGGCTATG	24

Table S3. Guides used to make single gene knockouts in HL-60s
The two highest performing guides from the genome-wide screen were chosen to make single gene knockouts in the HL-60 cell line. See Methods for details.

bioRxiv preprint doi: https://doi.org/10.1101/2023.05.15.540704; this version posted March 29, 2024. The copyright holder for this preprint (which was not certified by peer review) is the author/funder, who has granted bioRxiv a license to display the preprint in perpetuity. It is made available under aCC-BY 4.0 International license.

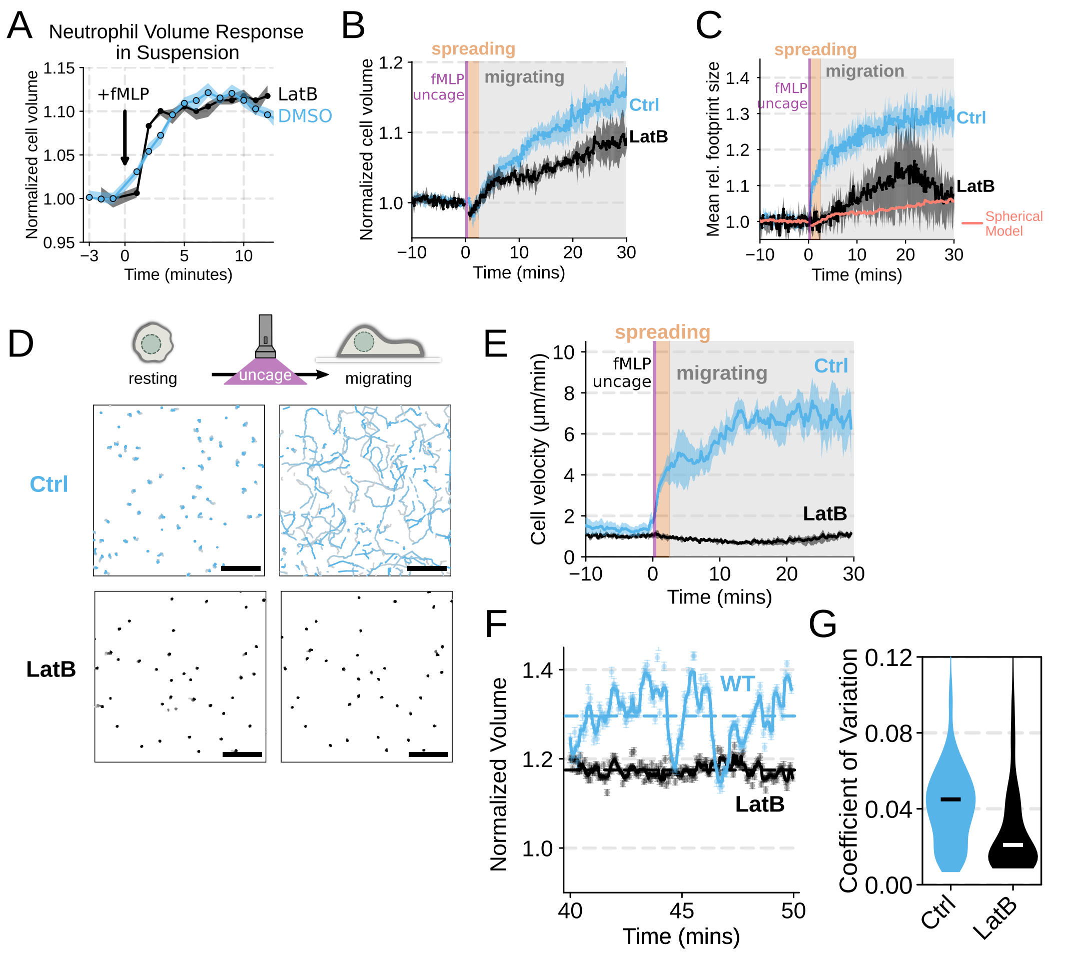

684

Figure 1—figure supplement 2. Chemoattractant-induced swelling, but not motility, is independent of actin polymerization.
(A) Human primary neutrophils were incubated with DMSO or Latrunculin B, activated with 20 nM fMLP, and then volume responses were measured using electronic sizing via a Coulter counter. Latrunculin treatment did not alter cell swelling, indicating that actin polymerization is dispensable for the chemoattractant-induced volume increase. **(B)** Similar results were obtained using the FxM assay, showing that Latrunculin-treated cells are capable of swelling after stimulation. **(C)** The Latrunculin-treated cells also increase their footprints, albeit less so than control cells, but this is within the range of what would be expected for this degree of chemoattractant-induced volume increase (modeled by a sphere expanding an equivalent volume). **(D)** Single cell tracks of primary human neutrophils responding to acute chemoattractant stimulation. Both panels show 15 minutes of tracks with the tracks prior (left) and the 15 minutes post (right) uncaging the chemoattractant. The scale bar is 50 microns. The top panels show the large increase in motility displayed by control cells, while the Latrunculin-treated cells (bottom panels) fail to move. See ***Figure 1—video 1*** for an animated version of this data. **(E)** Latrunculin-treated cells consistently fail to move in response to chemoattractant-stimulation. **(F)** Representative single cell volume traces show that Latrunculin-treated cells (black) lack short-term volume fluctuations but persistently maintain an elevated volume following chemoattractant stimulation. Control cells (blue) exhibit short-term volume fluctuations. **(G)** The lack of short-term volume fluctuations following latrunculin treatment is borne out across the population, with the coefficient of variation in the volume for single cells (post-swelling) being dramatically lower in Latrunculin-treated cells, suggesting that these short term volume fluctuations depend on actin-based motility.
Figure 1—figure supplement 2—source data 1. Raw and processed Fluorescence Exclusion Microscopy data available via DataDryad; see https://doi.org/10.7272/Q6NS0S5N

bioRxiv preprint doi: https://doi.org/10.1101/2023.05.15.540704; this version posted March 29, 2024. The copyright holder for this preprint (which was not certified by peer review) is the author/funder, who has granted bioRxiv a license to display the preprint in perpetuity. It is made available under aCC-BY 4.0 International license.

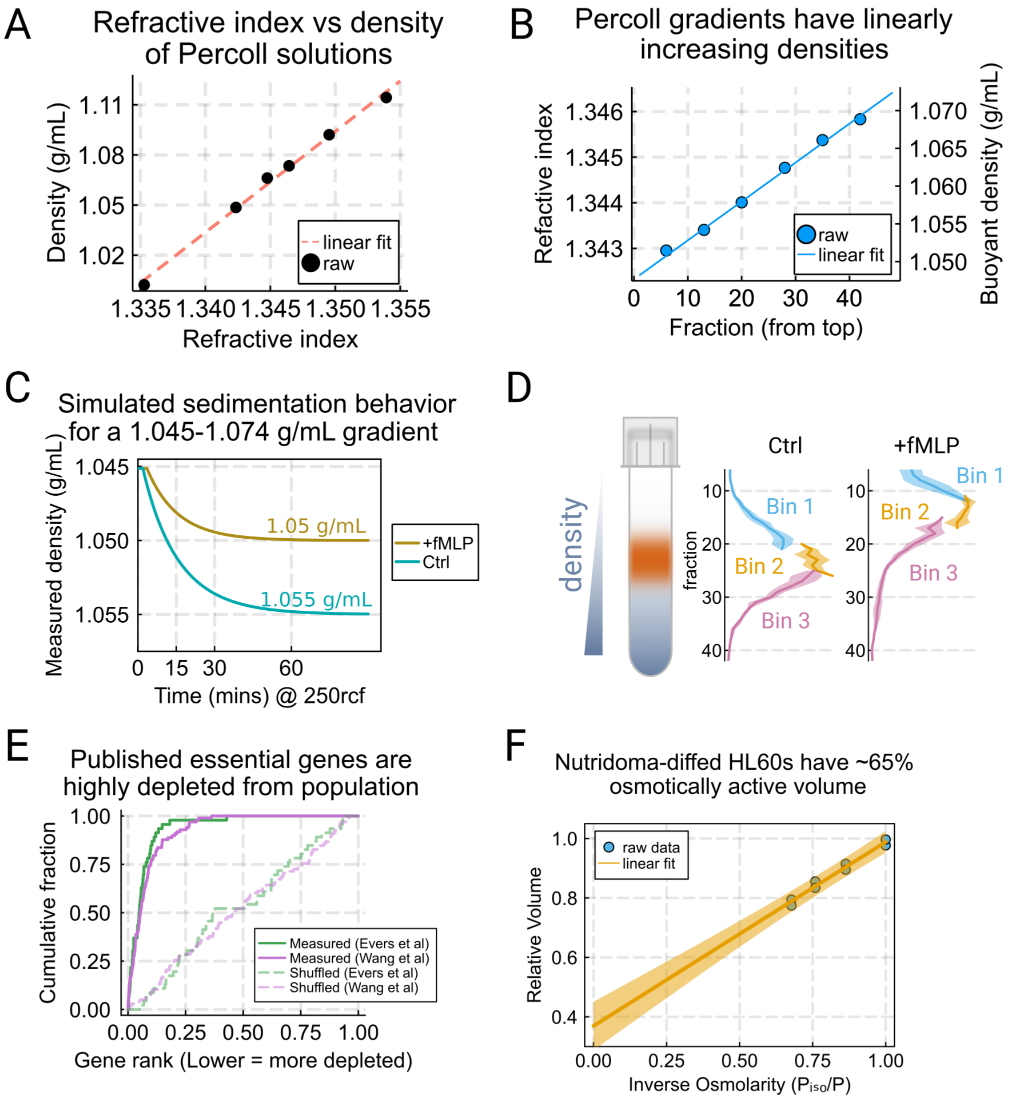

Figure 2—figure supplement 1. Validation of buoyant density assay and its use in CRISPR KO genome-wide screen for swelling regulators

(**A**) Varying the amount of Percoll shows the linear relationship between the refractive index (as measured by a refractometer) and the density as determined by weighing solutions in a volumetric flask on an analytical balance. (**B**) Measuring the refractive index of different fractions from the gradient shows a high degree of linearity in buoyant density. (**C**) Simulation of the settling behavior of the control (teal) and stimulated cells (gold) in the Percoll gradients under a centrifugal force of 250xg. The cells are predicted to arrive at their isopycnic point after approximately one hour. (**D**) The binning strategy for the screen for the control (left) and stimulated (right) conditions. For each replicate of each condition, the cells were split across 6 different tubes and these were combined into 3 bins to balance the minimum number of cells per bin with the resolution gained from multiple bins. (**E**) Previously published essential genes from ***Evers et al.*** (***2016***) and ***Wang et al.*** (***2015***), in green and purple, respectively, were highly depleted from the population, validating functionality of our CRISPR-based knockout library. (**F**) Ponder's relation for dHL-60s in suspension. Increasing amounts of hyperosmolarity drives the osmotically active fraction of the volume out of the cell, and projecting out to infinite osmolarity gives 65% of the cell volume as osmotically active.

bioRxiv preprint doi: https://doi.org/10.1101/2023.05.15.540704; this version posted March 29, 2024. The copyright holder for this preprint (which was not certified by peer review) is the author/funder, who has granted bioRxiv a license to display the preprint in perpetuity. It is made available under aCC-BY 4.0 International license.

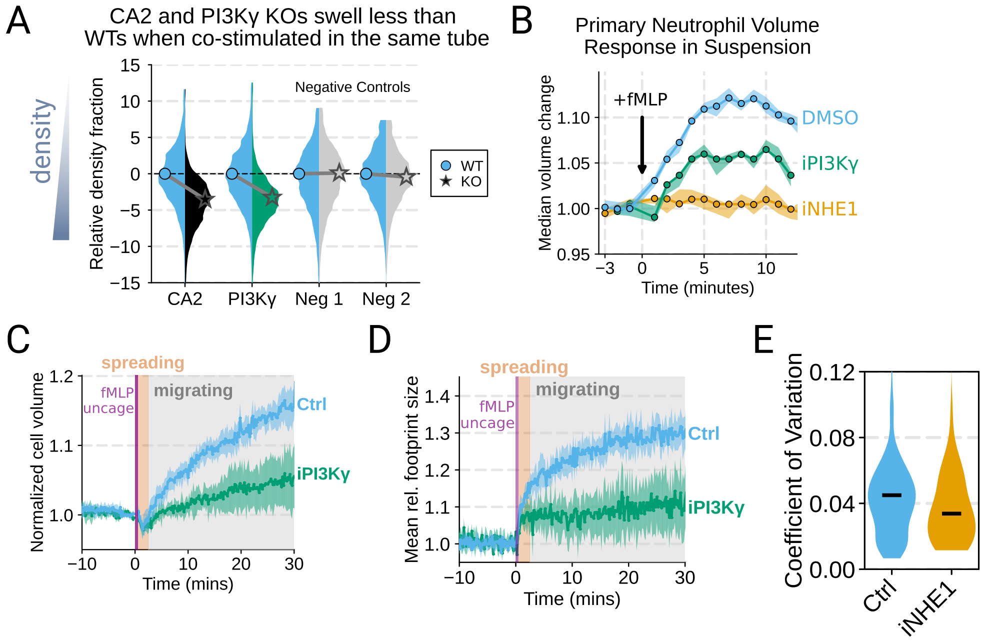

686

Figure 3—figure supplement 1. Additional validation of swelling screen hits.
(A) Mixed WT and CRISPR KO dHL-60 populations post-stimulation show that CA2 (black) and PI3Kγ (green) KO both fail to decrease their densities as much as the WT (cyan) population following chemoattractant stimulation. Cells with negative control guides (light gray) have normal volume responses. All tubes were fractionated and aligned on the fraction containing the median of the WT population. Negative values indicate a fraction with a higher density than WT. **(B)** To validate the perturbations to cell swelling observed with FxM, primary human neutrophils were stimulated in suspension, and their volumes were measured using a Coulter counter. 20 nM fMLP was added at the 0 minute mark. Shaded regions represent the 95% confidence intervals. **(C)** PI3Kγ inhibition blocks the chemoattractant-induced volume change in primary human neutrophils, as assayed by FxM. **(D)** PI3Kγ inhibition also blocked the chemoattractant-drive shape change in human primary neutrophils, as measured by the change in footprint area in FxM. **(E)** The coefficient of variation in volume for control (cyan) and iNHE1 (gold) inhibited human primary neutrophils undergoing chemokinesis are comparable, suggesting that the volume fluctuations are unchanged in moving cells upon NHE1 and PI3Kγ inhibition despite the different baseline volumes.

Figure 3—figure supplement 1—source data 1. Raw and processed FxM and Coulter data available via DataDryad; see https://doi.org/10.7272/Q6NS0S5N

bioRxiv preprint doi: https://doi.org/10.1101/2023.05.15.540704; this version posted March 29, 2024. The copyright holder for this preprint (which was not certified by peer review) is the author/funder, who has granted bioRxiv a license to display the preprint in perpetuity. It is made available under aCC-BY 4.0 International license.

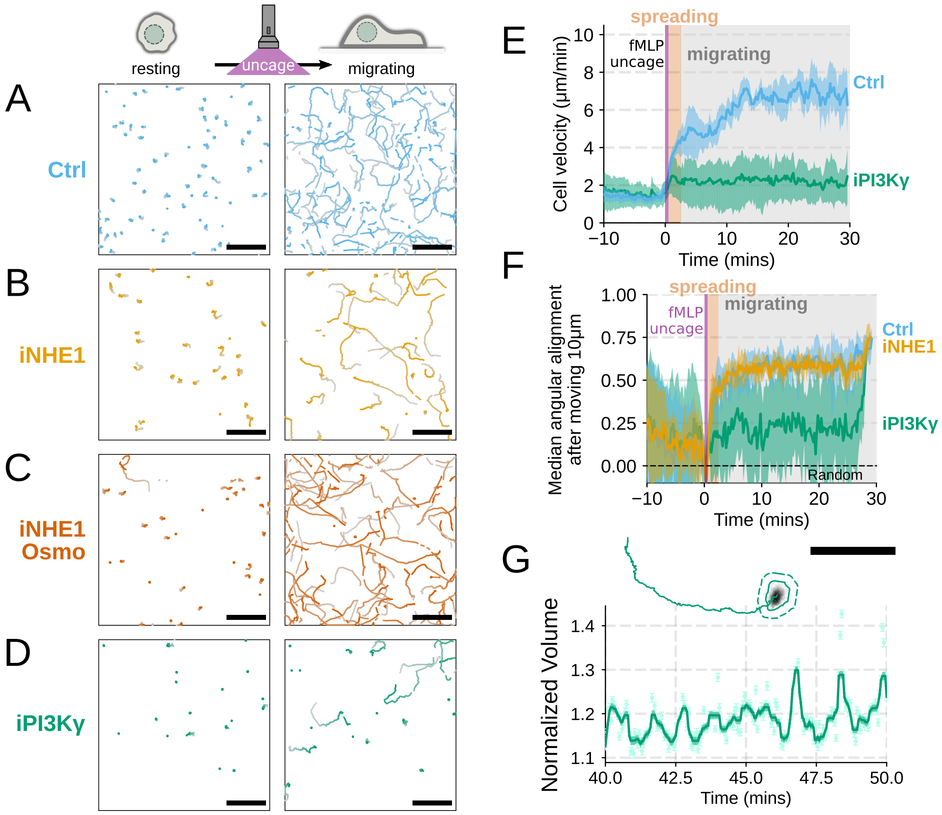

Figure 4—figure supplement 1. Additional validation of motility phenotypes.
(A-D) Single cell tracks of primary human neutrophils responding to acute chemoattractant stimulation. Both panels show tracks of cells 15 minutes prior (left) versus 15 minutes post (right) uncaging the chemoattractant. The scale bar is 50 microns. Color saturation indicates time with tracks progressing from gray to full color. Animated versions are available as ***Video 5*** and ***Figure 4—video 1***. **(A)** Control cells show a large increase in movement upon uncaging, **(B)** NHE1 inhibited cells also initiate movement but to a lesser degree, **(C)** hypo-osmotic shock rescues the NHE1 motility defect. **(D)** PI3Kγ leads to a large fraction of cells failing to initiate movement. **(E)** PI3Kγ inhibition showed near complete blockage of the chemoattractant-induced motility increase in primary human neutrophils. **(F)** Control neutrophils (blue) show an increased angular alignment upon stimulation as their motility becomes directional. NHE1-inhibition (gold, iNHE1) has very little effect on this process, while PI3Kγ inhibition (green) leads to a reduction in this alignment at the population level. **(G)** For the PI3Kγ inhibited cells that start migrating, the migration-induced volume fluctuations are comparable to iNHE1 and control cells. The top panel shows the track of a representative migrating PI3Kγ inhibited cell and the bottom panel, its corresponding volume normalized to the pre-stimulation volume. The scale bar is 50 microns.
Figure 4—figure supplement 1—source data 1. Raw and processed FxM data available via DataDryad; see https://doi.org/10.7272/Q6NS0S5N
