# Supplementary material for: Neutrophils actively swell to potentiate rapid migration
Source: bioRxiv. 2023 Jul 5:2023.05.15.540704. Originally published 2023 May 16. Preprint. [Version 2] doi: 10.1101/2023.05.15.540704 (PMC10245588; doi:10.1101/2023.05.15.540704)
Supplement: Supplementary file 1 [file NIHPP2023.05.15.540704v2-supplement-6.pdf]

bioRxiv preprint doi: https://doi.org/10.1101/2023.05.15.540704; this version posted July 5, 2023. The copyright holder for this preprint (which was not certified by peer review) is the author/funder, who has granted bioRxiv a license to display the preprint in perpetuity. It is made available under aCC-BY 4.0 International license.

Supplementary Information

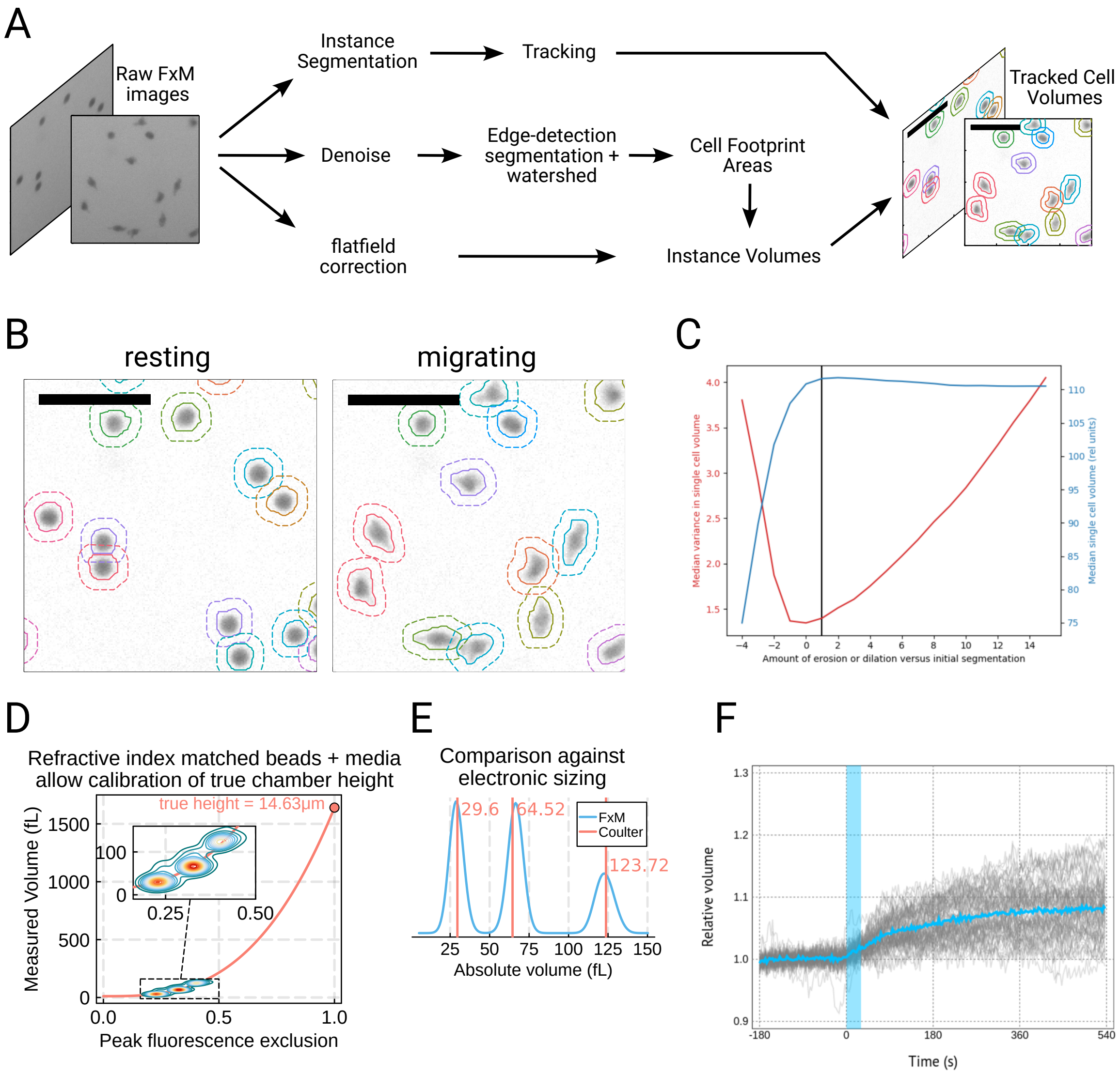

Figure S1. Details and validation of the Fluorescence eXclusion Microscopy pipeline

(**A**) Overview of the modified FxM pipeline used in this work. Raw FxM images are segmented into "seeds," which are then tracked. To identify cell boundaries, we use a custom algorithm that involves denoising the raw FxM followed by edge detection and watershedding that was nucleated at the seeds. Finally, to extract the volumes we apply minimal processing with a custom flatfield correction algorithm followed by extraction using the previously-determined cell boundaries. (**B**) Examples of resting (left) and migrating (right) cells segmented and tracked using the pipeline above (S1A). (**C**) Quantifying the signal-to-noise tradeoff with the edge detection. Overly conservative or overly liberal segmentation leads to non-optimal volume measurement. The chosen cutoff (black line) balances the noise (red) and measured volume (blue). This was computed on unstimulated cells. (**D**) Injecting beads into the FxM chambers in refractive-index media to minimize distortions allows for true calibration of chamber height. The contour plot of the per-bead max signal depth (i.e. height of the beads from FxM) vs the measured volume using FxM is well fit by the spherical volume formula (salmon line). The intersection of this line with 1.0 on the abscissa is equivalent to a sphere that completely fills the chambers and indicates the true height of the chamber. (**E**) Comparing calibrated FxM volumes of the beads (cyan) against the gold standard Coulter counter (salmon) shows good agreement. (**F**) dHL-60 cells treated with 1uM Latruculin-B swell in response to chemoattractant stimulation but lack cell spreading and spreading-related volume loss.

bioRxiv preprint doi: https://doi.org/10.1101/2023.05.15.540704; this version posted July 5, 2023. The copyright holder for this preprint (which was not certified by peer review) is the author/funder, who has granted bioRxiv a license to display the preprint in perpetuity. It is made available under aCC-BY 4.0 International license.

Ranking	HUGO ID	Fold Change	FDR	# Good sgRNAs	Common Name
**1**	**FPR1**	**4.4617**	**0.00248**	**3**	**FPR1**
2	STAT3	2.8815	0.00248	4	
**3**	**SLC9A1**	**3.5013**	**0.00495**	**4**	**NHE1**
**4**	**PIK3CG**	**3.3557**	**0.05569**	**4**	**PI3Kγ**
5	MARS2	23.6178	0.06374	3	
6	NUP35	14.5253	0.06374	3	
7	SKA1	3.3554	0.06374	4	
**8**	**CA2**	**2.8157**	**0.09942**	**4**	**CA2**
9	CANX	3.966	0.09942	4	
10	COPS3	8.2426	0.10015	4	
11	RPS2	18.7498	0.07976	3	
12	TPR	9.6024	0.16455	4	
13	RFT1	26.2401	0.16455	2	
14	SETD1A	11.1409	0.16455	2	
15	TP53TG3C	11.2083	0.16455	3	
16	UHRF1	4.7194	0.16455	3	
17	THG1L	10.4411	0.16455	3	
18	MRPL20	10.8506	0.16455	4	
19	UTP11L	9.0882	0.16455	3	
20	TPI1	17.976	0.16455	3	
21	CT47A6	2.8505	0.16455	3	
22	ESCO2	11.9249	0.1982	4	
23	GRXCR2	2.8457	0.1982	4	
24	PARP12	21.181	0.1982	2	
25	NR1H2	39.6651	0.20894	2	
26	PPP2R2A	4.4472	0.20894	4	
27	SLC25A26	20.7941	0.20894	2	
28	GANAB	2.7762	0.20894	3	
29	MMGT1	22.231	0.24088	2	
30	TCEB2	9.1192	0.26382	4	
31	MRPL17	9.465	0.26382	3	
32	C1QB	2.7157	0.26382	4	
33	TMX3	3.0917	0.26382	4	
34	TOPBP1	14.0471	0.16455	3	
35	AP3M1	7.9696	0.26528	2	
36	MSMB	4.5751	0.26528	4	
37	WDR83	5.4812	0.26528	4	
38	MARCH7	6.5514	0.2937	2	
39	RNF111	2.9736	0.2937	4	
40	MOGS	3.5169	0.2937	3	
**100**	**SLC4A2**	**2.582**	**0.54636**	**2**	**AE2**

Table S1. Chemoattractant-induced swelling genome-wide CRISPR KO screen hits
Rankings determined using MAGeCK Li et al. (2014). Fold change is the median fold enrichment in the dense bin versus the other two bins of the functional guides. FDR is the false discovery rate of each gene given the distribution of negative control guides in the library. See Methods section for details.

bioRxiv preprint doi: https://doi.org/10.1101/2023.05.15.540704; this version posted July 5, 2023. The copyright holder for this preprint (which was not certified by peer review) is the author/funder, who has granted bioRxiv a license to display the preprint in perpetuity. It is made available under aCC-BY 4.0 International license.

Volunteer	Age	Sex
4	27	M
5	30	M
6	37	F
8	31	M
14	24	M
17	29	F
18	28	M
19	30	M

Table S2. Volunteer Demographic Information
Demographic information collected for the volunteer donors according to the Institutional Review Board-approved study protocol at the University of California - San Francisco (Study #21-35147)

HUGO gene name	Takara sgRNA ID	Guide Sequence (5' -> 3')	Internal ID
FPR1	sgFPR1_2	CTACAGTACCTGGTAAAACG	11
FPR1	sgFPR1_3	CTGACAGCAACGATGGACAT	12
SLC9A1	sgSLC9A1_2	TTTGCCAACTACGAACACGT	13
SLC9A1	sgSLC9A1_4	TGAGGAACAGGTCACACATG	15
PIK3CG	sgPIK3CG_1	ACTTAACCCTCTCACAGCAG	16
PIK3CG	sgPIK3CG_3	GAGAATACGTCCTCCACATG	18
CA2	sgCA2_2	TATGAGTGTCGATGTCAACA	19
CA2	sgCA2_4	TCACTGGAACACCAAATATG	20
SLC4A2	sgSLC4A2_2	ACCTGCCCCACATACCCACA	21
SLC4A2	sgSLC4A2_3	GAAGACGCAGGACCTGATAG	22
NegCtrl	Neg_Control_Human_0067	GGTAGGACCTCACGGCGCGC	23
NegCtrl	Neg_Control_Human_0084	GCTGTCGTGTGGAGGCTATG	24

Table S3. Guides used to make single gene knockouts in HL-60s
The two highest performing guides from the genome-wide screen were chosen to make single gene knockouts in the HL-60 cell line. See Methods for details.

bioRxiv preprint doi: https://doi.org/10.1101/2023.05.15.540704; this version posted July 5, 2023. The copyright holder for this preprint (which was not certified by peer review) is the author/funder, who has granted bioRxiv a license to display the preprint in perpetuity. It is made available under aCC-BY 4.0 International license.

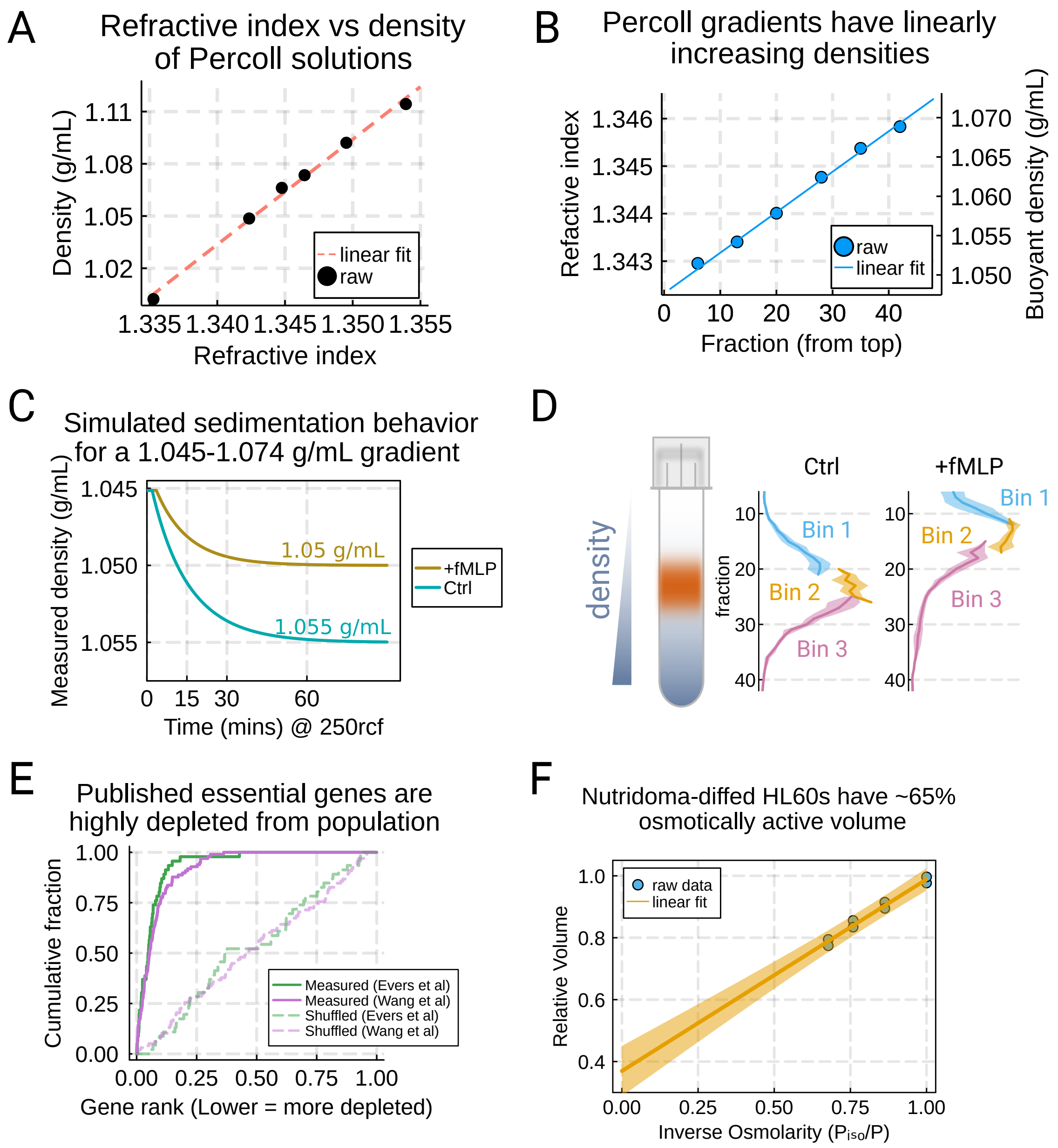

Figure S2. Validation of buoyant density assay and its use in CRISPR KO genome-wide screen for swelling regulators
(**A**) Varying the amount of Percoll shows the linear relationship between the refractive index (as measured by a refractometer) and the density as determined by weighing solutions in a volumetric flask on an analytical balance. (**B**) Measuring the refractive index of different fractions from the gradient shows a high degree of linearity in buoyant density. (**C**) Simulation of the settling behavior of the control (teal) and stimulated cells (gold) in the Percoll gradients under a centrifugal force of 250xg. The cells are predicted to arrive at their isopycnic point after approximately one hour. (**D**) The binning strategy for the screen for the control (left) and stimulated (right) conditions. For each replicate of each condition, the cells were split across 6 different tubes and these were combined into 3 bins to balance the minimum number of cells per bin with the resolution gained from multiple bins. (**E**) Previously published essential genes from Evers et al. (2016) and Wang et al. (2015), in green and purple, respectively, were highly depleted from the population, validating functionality of our CRISPR-based knockout library. (**F**) Ponder's relation for dHL-60s in suspension. Increasing amounts of hyperosmolarity drives the osmotically active fraction of the volume out of the cell, and projecting out to infinite osmolarity gives 65% of the cell volume as osmotically active.

bioRxiv preprint doi: https://doi.org/10.1101/2023.05.15.540704; this version posted July 5, 2023. The copyright holder for this preprint (which was not certified by peer review) is the author/funder, who has granted bioRxiv a license to display the preprint in perpetuity. It is made available under aCC-BY 4.0 International license.

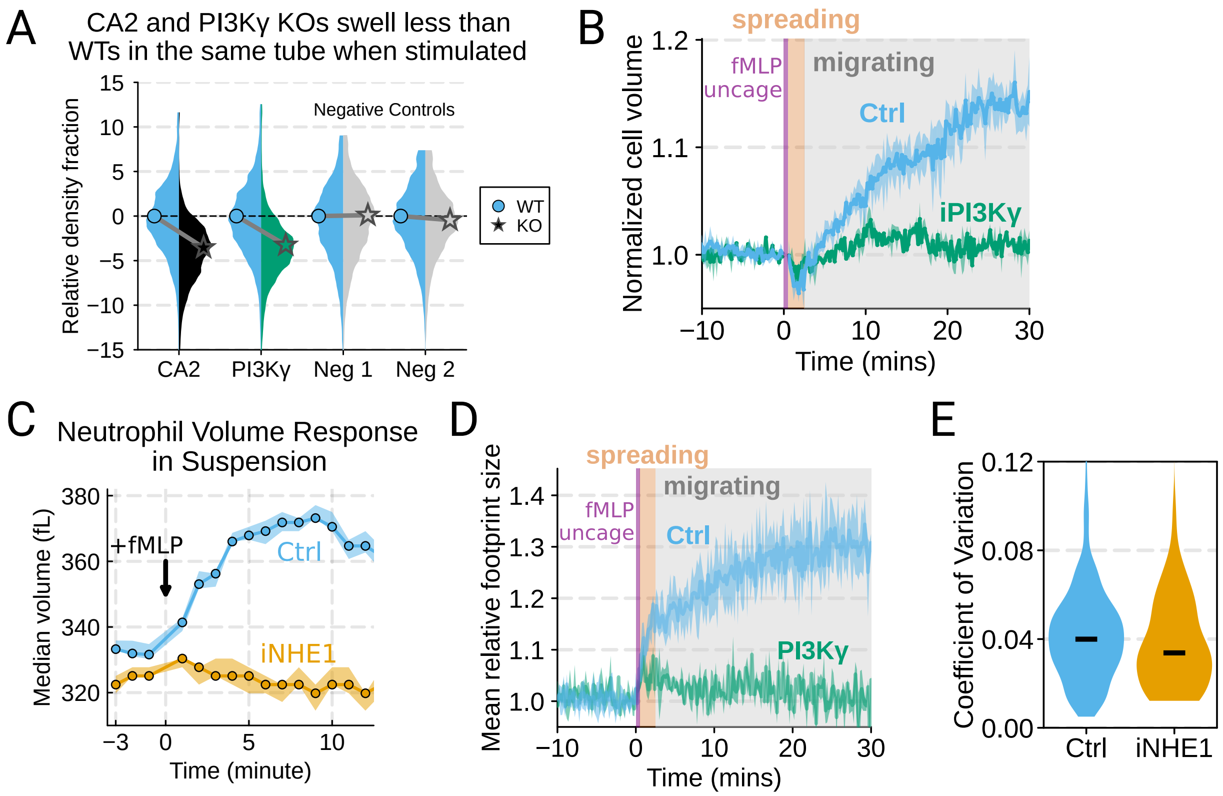

Figure S3. Additional validation of swelling screen hits

(**A**) Mixed WT and CRISPR KO dHL-60 populations post-stimulation show that CA2 (black) and PI3Kγ (green) KO both fail to decrease their densities as much as the WT (cyan) population following chemoattractant stimulation. Cells with negative control guides (light grey) have normal volume responses. (**B**) PI3Kδ/γ inhibition completely blocks the chemoattractant-induced volume change in primary human neutrophils. (**C**) To validate the FxM results, primary human neutrophils were stimulated in suspension, and their volumes were measured using a Coulter counter (WT replicates = 4, NHE1 = 2). 20 nM fMLP was added at the 0 minute marker. (**D**) PI3Kδ/γ inhibition blocked the chemoattractant-drive shape change in human primary neutrophils, as measured by the change in footprint area in FxM (**E**) The coefficient of variation in volume for control (cyan) and iNHE1 (gold) human primary neutrophils undergoing chemokinesis are comparable, suggesting that the volume fluctuations are unchanged upon NHE1 inhibition despite the different baseline volumes.

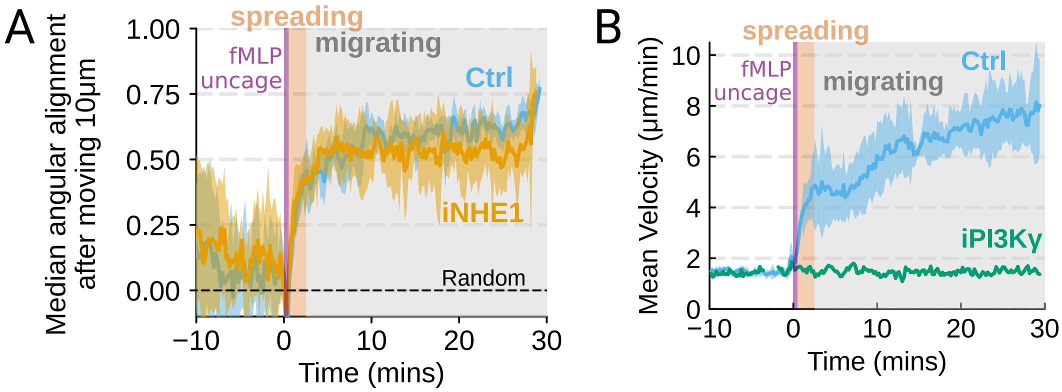

Figure S4. Additional validation of motility phenotypes

(**A**) Control neutrophils show an increased angular alignment upon stimulation as their motility becomes directional. NHE1-inhibition has very little effect on this process. (**B**) PI3Kδ/γ completely blocked the chemoattractant-induced motility increase in primary human neutrophils

bioRxiv preprint doi: https://doi.org/10.1101/2023.05.15.540704; this version posted July 5, 2023. The copyright holder for this preprint (which was not certified by peer review) is the author/funder, who has granted bioRxiv a license to display the preprint in perpetuity. It is made available under aCC-BY 4.0 International license.

Supplementary Videos

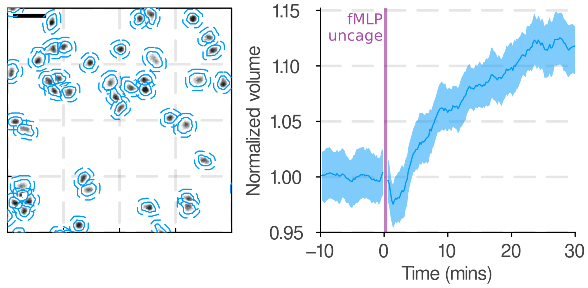

Video SV1. Primary human neutrophils show a biphasic volume response to chemoattractant stimulation
Left panel shows the individual cells (cyan solid lines) with their individual localities (dashed lines). The actual FxM signal is visible within the cell footprints. The movie is 40 minutes long with chemoattractant uncaging after the first 10 minutes. The scale bar is 50 um. The right panel shows the corresponding median volume of the population (cyan) with the 95% confidence interval (light cyan). The volume is relative to the average over the two minute window prior to uncaging.

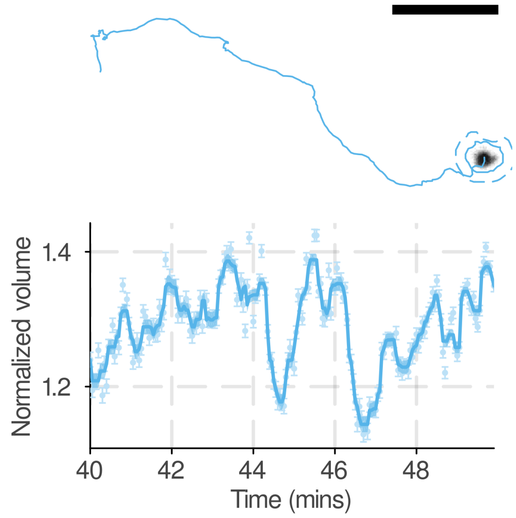

Video SV2. Single cell volume tracking reveals motility-associated volume changes in migrating primary human neutrophils
Top panel shows a representative cell migrating starting at 40 minutes following chemoattractant stimulation. The solid cyan line is the cell track. The cell's footprint is denoted with a solid cyan line while its local background is encapsulated by the dashed cyan line. The scale bar is 50 um. The bottom panel shows the concurrent volume changes with the raw FxM measurements displayed in light cyan and the rolling median is shown in cyan. The volume is normalized to the median cell volume in the two minute window prior to stimulation.

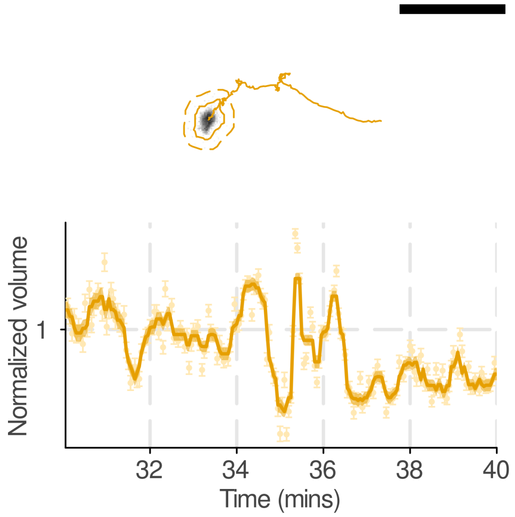

Video SV3. NHE1-inhibited primary human neutrophils retain the motility-related volume fluctuations but at much lower baseline volumes
Top panel shows a representative cell migrating starting at 30 minutes following chemoattractant stimulation. The solid gold line is the cell track and the cell's footprint is denoted with a solid gold line while its local background is encapsulated by the dashed gold line. The scale bar is 50 um. The bottom panel shows the concurrent volume changes with the raw FxM measurements displayed in light gold and the rolling median is shown in gold. The volume is normalized to the median cell volume in the two minute window prior to stimulation.

bioRxiv preprint doi: https://doi.org/10.1101/2023.05.15.540704; this version posted July 5, 2023. The copyright holder for this preprint (which was not certified by peer review) is the author/funder, who has granted bioRxiv a license to display the preprint in perpetuity. It is made available under aCC-BY 4.0 International license.

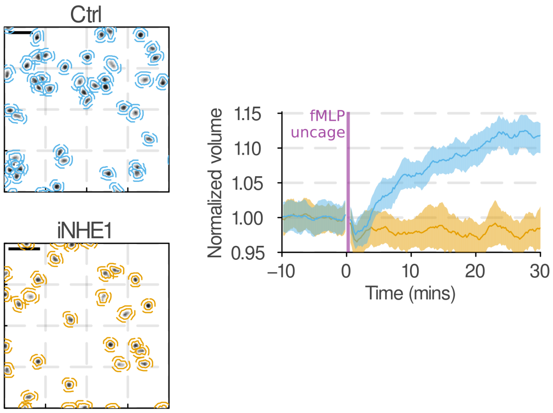

Video SV4. NHE1 inhibition blocks chemoattractant-induced cell swelling and impairs chemokinesis
Left panel shows the individual cells (cyan or gold solid lines) with their individual localities (dashed lines). The top left panel are control cells (cyan) and the bottom left are NHE1 inhibited (gold). The actual FxM signal is visible within the cell footprints. The movie is 40 minutes long with chemoattractant uncaging after the first 10 minutes. The scale bars are 50 um. The right panel shows the corresponding median volume of the population in cyan and gold for control and iNHE1, respectively. The corresponding 95% confidence interval is in light cyan or gold. The volume is relative to the average over the two minute window prior to uncaging.

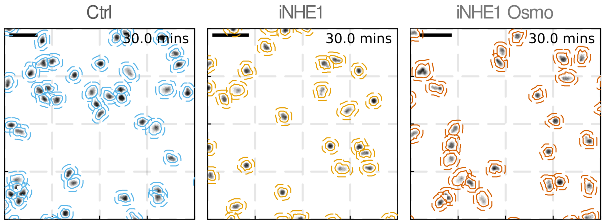

Video SV5. NHE1-inhibition leads to impaired motility versus control, which is rescued by mild hypoosmotic shock
Each panel is a representative video of control, NHE1-inhibited, and hypoosmotically shocked NHE1-inhibited cells, respectively. Each video is 40 minutes long and chemoattractant uncaging occurs after 10 minutes. Scale bars are 50 um.
